# The relative timing of VMO and VL in the aetiology of anterior knee pain: a systematic review and meta-analysis

**DOI:** 10.1186/1471-2474-9-64

**Published:** 2008-05-01

**Authors:** Rachel Chester, Toby O Smith, David Sweeting, John Dixon, Sarah Wood, Fujian Song

**Affiliations:** 1 School of Allied Health Professions, University of East Anglia, Norwich, Norfolk, NR4 7TJ, UK; 2 Physiotherapy Department, Norfolk and Norwich University Hospital, Norwich, NR4 7UY, UK; 3 Physiotherapy Department, Great Yarmouth & Waveney PCT, James Paget University Hospital NHS Trust, Great Yarmouth, Norfolk, NR31 6LA, UK; 4 Centre for Rehabilitation Sciences, School of Health and Social Care, University of Teesside, Middlesbrough, TS1 3BA, UK

## Abstract

**Background:**

Anterior knee pain (AKP) is a common musculoskeletal complaint. It has been suggested that one factor that may contribute to the presence of AKP is a delay in the recruitment of the vastus medialis oblique muscle (VMO) relative to the vastus lateralis muscle (VL). There is however little consensus within the literature regarding the existence or nature of any such delay in the recruitment of the VMO within the AKP population. The purpose of this systematic review and meta-analysis was to examine the relative timing of onset of the VMO and VL in those with AKP in comparison to the asymptomatic population.

**Methods:**

The bibliographic databases AMED, British Nursing Index, CINAHL, EMBASE, Ovid Medline, PEDro, Pubmed and the Cochrane Library were searched for studies comparing the timing of EMG onset of the VMO and VL in those with AKP versus the asymptomatic population. Studies fulfilling the inclusion criteria were independently assessed. Heterogeneity across the studies was measured. A meta-analysis of results was completed for those studies where adequate data was supplied. Where comparable methodologies had been used, results were pooled and analysed.

**Results:**

Fourteen studies met the inclusion criteria; one prospective and thirteen observational case control. Eleven compared VMO and VL EMG onset times during voluntary active tasks while four investigated reflex response times. All used convenience sampling and did not state blinding of the assessor. Study methodologies/testing and assessment procedures varied and there was considerable heterogeneity within individual samples. Whilst a trend was identified towards a delay in onset of VMO relative to the VL in the AKP population during both voluntary active tasks and reflex activity, a substantial degree of heterogeneity across the pooled studies was identified (I^2 ^= 69.9–93.4%, p < 0.01).

**Conclusion:**

Findings are subject to substantial and unexplained heterogeneity. A trend was demonstrated towards a delayed onset of VMO relative to VL in those with AKP in comparison to those without. However not all AKP patients demonstrate a VMO-VL dysfunction, and this is compounded by normal physiological variability in the healthy population. The clinical and therapeutic significance is therefore difficult to assess.

## Background

Anterior knee pain (AKP) is one of the most common conditions presenting to physiotherapists [1], with a reported incidence in up to 25% of the population [2]. Despite this, the exact cause of pain remains largely unknown [3]. It has been suggested that a variety of factors may contribute to the development and maintenance of AKP. One such factor is the presence of a delay in the recruitment of the vastus medialis oblique muscle (VMO) relative to the vastus lateralis muscle (VL) during functional activity [2,4]. It has been claimed that the presence of such a delay may adversely affect the tracking of the patella, thus contributing towards the presence of AKP [2,4-6]. One objective of some treatment strategies commonly used in the rehabilitation of AKP is to restore the normal timing of the VMO and VL muscles [2,4].

Previous descriptive reviews however have highlighted potential disagreements within the literature regarding the scale, existence and even the direction of any abnormality in the firing patterns of VMO and VL in the AKP population [7,8]. This brings into question the validity of the basis upon which these treatment strategies are based. Improved understanding regarding the existence and nature of any dysfunction in the timing of these muscles would contribute to the effective management of this common condition. The purpose of this systematic review is to synthesise evidence from comparative studies that have investigated the relative onset timing of the VMO and VL in AKP and asymptomatic control subjects.

## Methods

### Search Strategy

The literature search was performed by TS using the bibliographic electronic databases AMED, British Nursing Index, CINAHL, EMBASE, Ovid Medline, Physiotherapy Evidence Database (PEDro), Pubmed and the Cochrane Library, from their inception to June 2006. The following Medical Subject Headings and key words were combined: anterior knee pain or patellofemoral pain syndrome or chondromalacia or extensor mechanism or vastus medialis or vastus lateralis; AND activity or timing or recruitment or torque or EMG or electromyography or electromyographic. This was updated by a second electronic search (DS and JD) for any publications in the period June 2006–June 2007.

A hand search was performed in the specialist journals: The Knee (1994- June 2006), Physical Therapy in Sport (2000- June 2006), American Journal of Sport Medicine (1986- June 2006), British Journal of Sport Medicine (1986- June 2006) and the Journal of Science and Medicine in Sport (1998- June 2006). The reference lists of all retrieved papers including review articles were searched for any additional publications unidentified by the initial search strategy.

### Study selection

We included any primary studies that compared differences in the onset timing in milliseconds (ms) of VMO and VL as a primary or secondary outcome between subjects with anterior knee pain, patellofemoral pain syndrome or chondromalacia patellae and asymptomatic control subjects. Studies which compared timing of peak EMG activity or percentage of the gait cycle were excluded as were those which included animals and cadavers or subjects suffering patellar instability. Full text English language publications only were included, regardless of the year of publication.

**Figure 1:**
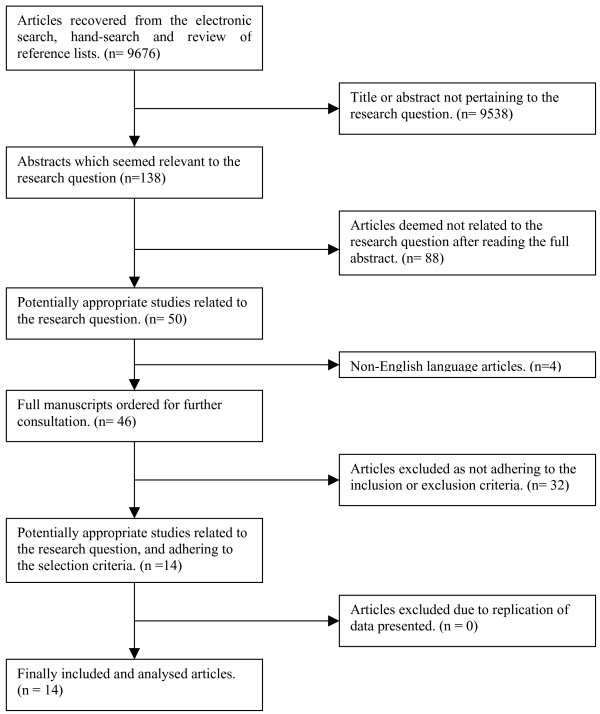
QUORUM flow chart.

Three reviewers independently (RC, DS and SW) screened the titles and abstracts of all identified papers to determine those potentially relevant to the review. The full manuscripts were then retrieved and each paper independently assessed for inclusion/exclusion criteria by two of four reviewers (RC, TS, DS, and SW), any doubts or disagreements were discussed between the four reviewers until a consensus was reached. The QUORUM flow chart (Figure 1) illustrates the process by which manuscripts were selected and numbers involved.

### Data extraction

Each study which met the inclusion criteria was independently assessed by two of four reviewers, (DS, RC, SW and TS), each of whom completed a data extraction form [see additional file 1]. This included: study design, participant selection, sample size, population characteristics of AKP subjects and control groups, procedural details and methods of EMG assessment, results, and relevant study limitations. Data extraction forms were compared for accuracy and interpretation; where there was disagreement or information was ambiguous all four reviewers met to reach an agreement. In the absence of a recognised methodological scoring system for comparative observational studies, a qualitative critical appraisal of each study was undertaken. This included an assessment of the factors identified from the data extraction form and their impact on the results, their interpretation and generalisability.

### Evidence synthesis and statistical methods

In relation to the relative timing of the VMO and VL, the most relevant and commonly used outcome measure was the onset timing difference (milliseconds) between the VMO and VL (i.e., *Δ *= *VMO-VL*). Where *Δ *> 0 indicates that the VMO onset was later than the VL onset, and *Δ *< 0 indicates that the VMO onset was earlier than the VL onset. In this systematic review, we compared the difference in relative timing of the VMO and VL between AKP patients and control subjects. That is, the primary outcome measure used in the meta-analyses was the mean difference (*MD*) between *Δ*_*AKP *_and *Δ*_*CTRL*_, where *Δ*_*AKP *_refers to the VMO-VL difference in AKP patients and *Δ*_*CTRL *_refers to the VMO-VL difference in control subjects. When *MD = 0*, it indicates that there was no difference in the onset timing of VMO relative to VL between AKP patients and control subjects. When *MD *> *0*, the onset of VMO was relatively later in AKP patients than that in control subjects.

Some primary studies did not provide sufficient data or statistics to allow meta-analysis. For several studies [5,6,15,20], we acquired data based on graphical illustration in published papers. Particularly, there was a lack of data on standard deviations of VMO-VL difference in AKP patients and VMO-VL difference in control subjects required for meta-analysis. An average estimate of standard deviation was therefore calculated based on data from other relevant studies [9] and input to studies in which the standard deviation was not available.

Heterogeneity in results across primary studies was statistically tested and measured by I^2 ^statistic [10]. Meta-analysis was carried out using REVMAN software (version 4.2 for Windows. Copenhagen: The Nordic Cochrane Centre, The Cochrane Collaboration, 2003). The existence of statistically significant heterogeneity means that the pooling of results from primary studies in meta-analysis may be controversial. When judged appropriate and helpful, we conducted meta-analyses using random-effects model. Significant heterogeneity was further narratively investigated in the discussion, by examining whether differences in study results could be possibly explained by different study characteristics. The possibility of publication bias in meta-analyses was statistically tested by using funnel-plot related methods, Begg's test [11] and Egger's test [12].

## Results

### Study Characteristics

A total of fourteen studies adhered to the pre-determined inclusion and exclusion criteria and were included in the review; eleven studies compared VMO and VL onset times during active and functional tasks, whilst four studies investigated reflex response times of VMO and VL during the patella tendon reflex reaction. Study design and methodological issues are presented in table 1 and population characteristics and procedural details are presented in table 2 and 3.

**Table 1:** Study design and methodological issues

Study/country of origin	Study design: Com.obs Pros/long	Subject Select-ion	Control selection: matched/NS	Justification. Sample size: Y/NS	Elect. position reprod: Y/N	Reliability assessed Y/N	Tester blind to group alloc: Y/NS	Sufficient results in text: Y/N	
Boling et al [15] USA	Com.obs	Conv	NS	NS	Y	Y	NS	Y
Brindle et al [21] USA	Com.obs	Conv	Matched for age	NS	N	N	NS	Y
Cowan et al [5] Australia	Com.obs	Conv	NS	NS	Y	Y	NS	N
Cowan et al [16] Australia	Com.obs	Conv	NS	From prev. study	Y	Y	NS	N
Cowan et al [20] Australia	Com.obs	Conv	Matched for gender	NS	Y	Y	NS	N
Crossley et al [14] Australia	Com.obs	Conv	NS	NS	N	Y	NS	Y
Earl et al [17] USA	Com.obs	Conv	Matched on no. of factors	NS	Y	Y	NS	N
Karst and Willett [30] USA	Com.obs	Conv	NS	NS	N	Y	NS	Y
McClinton et al [22] USA	Com.obs	Conv	NS	NS	Y	Y	NS	Y
Morrish & Woled ge [46] UK	Com.obs	Conv	NS	NS	N	N	NS	N
Owings et al [19] USA	Com.obs	Conv	NS	NS	N	N	NS	Y
Witvrouw et al [18] Belgium	Com.obs	Conv	NS	NS	Y	Y	NS	Y
Witvrouw et al [13] Belgium	Pros. long.	Conv	NA	Not specifically for timing	Y	Y	NS	Y
Voight & Wieder [6] USA	Com.obs	Conv	NS	NS	N	N	NS	N

Abbreviations: Com.obs – Comparative observational, Pros. long – Prospective longitudinal study. Conv – convenience, NS – not stated, NA – Not applicable, Y – yes, N – No, Reprod – Reproducible

**Table 2:** Population characteristics and procedural details. Voluntary muscle activation

Study and country of origin	Subject details (age in years; height in cm)	Asymptomatic control details (age in years; height in cm)	Task	Electrode type; sampling rate; signal processing	EMG onset determination method	AKP during test
Boling et al [15] USA	No start/completion: 14/14 M/F: 5/9 Age: 24 ± 6 Height: 167.5 ± 10.1 Profile: University population and local community. Symptom Duration: Min 2 months	No start – completion: 14/14 M/F: 5/9 Age: 23 ± 2 Height: 170.9 ± 7.3 Profile: University population.	Stance phase asc/desc stair (20 cm step height)	Surface Electrodes; SR 1000 Hz; FWR & LPF 50 Hz	Point at which signal exceeded mean + 3 SD of baseline level for minimum of 25 ms	NS
Brindle et al [21] USA	No: 16/16 M/F: 4/12 Age: range 18–35 Height: NS Profile: Student population and local community. Symptom Duration: Min 2 months	No: 12/12 M/F: 5/7 Age: "Age matched" Height: NS Profile: NS	Stance phase asc/desc stairs (18 cm step height, 22 cm depth)	Surface Electrodes; SR 960 Hz; FWR & LPF 15 Hz	Point at which mean voltage of a moving 25 ms window exceeded mean + 5 SD of baseline level	Yes. 4.4 ± 3.0 during asc., 4.25 ± 1.8 during desc (on 10 cm VAS)
Cowan et al [5] Australia	No: 33/33 M/F: 11/22 Age: 27.0 ± 8.1 Height: 171.1 ± 9.3 Profile: NS Symptom Duration: 42.2 months ± 49.9 (1–144)	No: 33/33 M/F: 13/20 Age: 23.6.0 ± 4.9 Height: 169.8 ± 11.9 Profile: University of Melbourne School of Physiotherapy.	Stance phase asc/desc stairs (20 cm step height)	Surface Electrodes; SR 1000 Hz; FWR & LPF 50 Hz	Point at which signal exceeded mean + 3 SD of baseline level for minimum of 25 ms	Yes 2.6 ± 2 (on 0–10 cm VAS)
Cowan et al [16] Australia	No: 10/10 M/F: 3/7 Age: 22.7 ± 8.0 Height: 167.1 ± 9.6 Profile: N/S Symptom duration: > 1 month	No: 12/12 M/F: 4/8 Age: 19.5 ± 1.4 Height: 170.9 ± 10.5 Profile: Students – School of Physiotherapy	Stance phase asc/desc stairs (20 cm step height)	Surface Electrodes; SR 1000 Hz; FWR & LPF 50 Hz	Point at which signal exceeded mean + 3 SD of baseline level for minimum of 25 ms	Yes 3.5 ± 0.5 (on 0–10 cm VAS)
Cowan et al [20] Australia	No: 37/37 M/F: 14/23 Age: 28.5 ± 7.3 Height: 170.7 ± 8.9 Profile: N/S Symptom duration: 10.9 months ± 22.3	No: 37/37 M/F: 14/23 Age: 24.4 ± 5.8 Height: 171.9 ± 12 Profile: Students – School of Physiotherapy	Reaction time to visual instruction – 2 random tasks: rocking back on heels or rising on toes	Surface Electrodes; SR 1000 Hz; FWR & LPF 50 Hz	Point at which signal exceeded mean + 3 SD of baseline level for minimum of 25 ms.	No
Crossley et al [14] Australia	No: 48/47 M/F: 17/31 Age: 28.5 ± 8 Height: 170 ± 9 Profile: N/S Symptom duration: 8 months ± 8	No: 18/18 M/F: 9/9 Age: 35.5 ± 5 Height: 172 ± 12 Profile: Students University of Melbourne	Stance phase asc/desc stair (20 cm step height)	Surface Electrodes; SR 1000 Hz; FWR & LPF 50 Hz	Point at which signal exceeded mean + 3 SD of baseline level for minimum of 25 ms.	Yes, 2.5 ± 2.0 (0–10 VAS)
Earl et al [17] USA	No: 16/15 M/F: 3/13 Age: 21.5 ± 4.2 Height: 165.3 ± 10.2 Profile: Recreational athletes. Physical therapy and Sports Medicine Clinics Symptom duration: 9.3 weeks, (1–24 weeks)	No: 16/15 M/F: 3/13 Age: 21.1 ± 11.5 Height: 165.6 ± 11.5 Profile: Recreational athletes matched on gender, age, height, weight and exercise per week.	Lateral step down off 20.3 cm block	Surface Electrodes; SR 1200 Hz; processed with RMS 10 data point window	Point at which signal exceeded mean + 3 SD of baseline level for minimum of 25 ms.	NS
Karst and Willett [30] USA	No: 24/24 M/F: 6/18 Age: 28.3 ± 7.6 Height: 172.0 ± 10.9 Profile: NS Symptom duration: At least one year	No: 24/24 M/F: 8/16 Age: 28.8 ± 7.9 Height: 173.5 ± 8.2 Profile: NS	1) See table below 2) NWB active knee extension 3) WB knee extension – lateral step up onto 8 cm block	Surface Electrodes; SR 4000 Hz; unclear what smoothing process used (e.g. LPF or RMS)	Point at which signal exceeded mean + 1 SD of baseline level	NS
McClinton et al [22] USA	No: 20/20 M/F: 11/9 Age: 29.5 ± 10.0 Height : 173 ± 10 Profile: NS Symptom duration: NS	No: 20/20 M/F: 10/10 Age: 25.4 ± 3.1 Height: 172 ± 12 Profile: NS	Stance phase asc stairs (8, 14, 20, 26, 32 cm step height)	Surface Electrodes; SR 960 Hz; FWR & LPF 6 Hz	Point at which signal exceeded + 2SD of baseline level for 20 ms.	Yes 1.7 ± 0.3 (14 cm step) to 3.1 ± 0.5 (32 cm step) (0–10 VAS)
Morrish and Woledge [46] UK	No: 49/49 M/F: 16/33 Age: 20–37 (median 26) Height: NS Symptom duration: 6–120 months. Profile: NS	No: 20/20 M/F: 7/13 Age: 20–33 (median 25) Height-not stated. Profile: University staff and students who were non-or recreational athletes.	Isometric knee extension in sitting, with knee in 20 degrees flexion.	Surface Electrodes; SR not stated; FWR & integrat'n over 100 ms intervals	Actual onset not determined. Level of EMG signal over time relative to quadriceps force generation (termed lag factor).	NS.
Owings et al [19] USA	No: 20/20 M/F: 8/12 Age: F33.7 ± 6.9; M29.1 ± 10.7 Height: F1.65 ± 0.06; M1.77 ± 0.08 Profile: NS Symptom Duration: NS	No: 14/14 M/F: 10/4 Age: F22.3 ± 1.6; M24.5 ± 2.3 Height: F1.63 ± 0.07, M1.81 ± 0.06 Profile: NS	Isokinetic knee flexion/extension in sitting. Hip and trunk angle approx 100 degrees. Control 60 degree/sec, Subjects 15 deg/sec.	Surface Electrodes; SR 1000 Hz; raw signal used for onset	Point at which signal surpassed resting value	NS but did need to change method due to pain.

Abbreviations:

AKP – anterior knee pain; approx – approximately; asc – ascending; cm – centimetres; deg – degrees; desc – descending; EMG – electromyography; F – Female; FWR – full wave rectification; LPF – low pass filter; M – Male; ms – milliseconds; No – Number; NS – Not stated; NWB – non weight bearing; RMS – root mean square; SD- standard deviation; sec – seconds; SR – sampling rate; VAS – visual analogue scale; WB – weight bearing

**Table 3:** Population characteristics and procedural details. Reflex activation.

Study and country of origin	Subject details (age in years; height in cm)	Asymptomatic control details (age in years; height in cm)	Task	Electrode type; sampling rate; signal processing	EMG onset determination method	AKP during test
Karst and Willett [30] USA	See table 1	See table 1	Reflex knee extension elicited by patella tendon tap. High sitting with leg hanging over side of plinth, hip and knee at 90 degrees flexion	Surface Electrodes: SR 4000 Hz; raw signal used	Computerized: point at which signal exceeded mean + 1 SD of baseline level	NS
Witvrouw et al [18] Belgium	No: 19/19 M/F:8/11 Age: 21.1, range 17–26 Height: NS Profile: University Hospital clinic, Dept of Orthopaedic Surgery. Symptom Duration: > 1 and 1/2 months	No: 80/80 M/F: 37/43 Age: 18.4, range 17–22 Height: NS Profile: NS		Surface Electrodes: SR not stated; raw signal used	Visual: point at which EMG leaves baseline.	NS
Witvrouw et al [13] Belgium	No: 480/282. 24 developed AKP and became subjects. Profile (includes control): Students taking physical education classes. 12–14 hrs sport per week. No history of AKP at start. M/F: 11/13 Mean age 18.6, (17–21) includes controls. Height: 179.3 ± 5.38 Symptom Duration: > 1 and 1/2 months	As across. 258 did not develop AKP and became controls. M/F: 151/131 Height: 180.16 ± 6.25		Surface Electrodes: SR not stated; raw signal used	Visual: point at which EMG leaves baseline.	NS
Voight and Wieder [6] USA	No: 16/16 M/F: 10/6 Age: 26.1 (19–31) Height: NS Profile:Professional, recreational and non-athletes from sports clinic Miami University. Symptom Duration: NS	No: 41/41 M/F: 17/24 Age: 24.8, (18 – 45) Height: NS Profile: "@age matched", otherwise NS		Surface Electrodes: SR not stated; raw signal used	Visual: point at which EMG leaves baseline.	NS

Abbreviations:

AKP – anterior knee pain; cm – centimetres; EMG – electromyography; F – female; FWR – full wave rectification; LPF – low pass filter; M – male; No – number; NS – not stated; RMS – root mean square; SD- standard deviation

Thirteen of the studies were comparative observational/case-control designs involving a total of 322 AKP subjects and 341 controls. The number of participants within each study ranged from 22 [16] to 74 [20]. One study [12] was a prospective longitudinal study with a two year follow-up of 282 students, 24 of whom developed AKP and became subjects; the remaining 258 acting as controls.

With the exception of the control group in Crossley et al's [13] study, the mean age of participants was thirty years and under, and in four studies, below 25 years [14-17]. Documented age ranges and calculations of two standard deviations from the mean indicated that over 95% of participants were above seventeen years of age and under forty five years of age, with the exception of the participants in two studies [6,18] where the upper age limit reached forty five years.

The duration of AKP pain was documented in eleven studies and varied considerably, ranging from one week [16] to 12 years [5]. Five studies did not indicate the presence or absence of AKP during testing. One study implied that AKP was experienced during testing [19], one clearly stated that AKP was not present during testing [20] and six gave full details of intensity [5,13-15,21,22].

All thirteen comparative observation/case control studies appeared to use convenience sampling, at least in part, by means of recruiting subjects and/or controls. The potential for examiner bias was not controlled in any of the papers reviewed; none of the studies indicate whether or not the researcher was blinded to group allocation during data collection or analysis. Electrode position was reproducible in eight of the fourteen studies. The number of tests prior to data collection, and whether results were recorded as a single test or mean of several, varied within the papers reviewed. Ten studies indicated that reliability was assessed. There were no studies which provided justification for the selected sample size and six studies did not provide complete results (mean and standard deviation of VMO-VL of each group). See table 1 for further details.

Differences between the tasks meant that it would be inappropriate to make any direct comparisons between the included studies. Six studies however investigated EMG onset during step ascent, and five studies during step descent, with steps ranging from 17.8–20.3 cm in height. Four studies investigated the patella tendon reflex reaction. The results for each of these three procedures were pooled for exploratory data analysis.

### Relative onset timing of the VMO and VL

**Figure 2:**
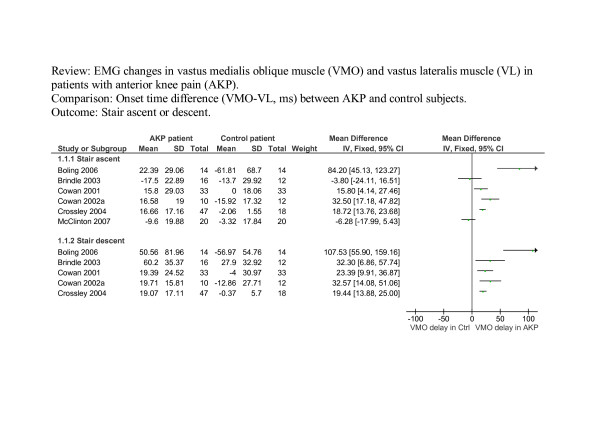
**The results of meta-analysis of studies investigating stair ascent and descent**. A negative VMO-VL value indicates VMO activation before VL activation. 2. Data for the mean and standard deviation was extracted from charts rather than the text for Cowan et al [5, 20] and standard deviation for Cowan et al [16]. 3. Standard deviation for Brindle et al [21] is an average estimate using data from other studies. 4. Small study bias: Stair ascent studies: Begg's test p = 0.71; Egger's test p = 0.40. Stair descent studies: Begg's test p = 0.09, Egger's test p = 0.04.

Figure 2 shows the results of meta-analysis of studies investigating stair ascent and descent. It may be of interest to note that the evidence presented in Figure 2 was mainly from a single research team (two studies by Cowan et al [5,16], and one by Crossley et al [14]).

There is substantial heterogeneity across the six studies investigating timing during stair ascent (I^2 ^= 85.7%, p < 0.00001) and across the five studies investigating stair descent (I^2 ^= 69.9%, p = 0.01). Boling et al [15] reported the greatest effect size (Figure 2). The test of small study bias (or funnel-plot asymmetry) using Begg's test and Egger's test was not statistically significant for studies of stair ascent (p = 0.71 and p = 0.40 respectively), but statistically significant for studies of stair descent by Egger's test (p = 0.09 and p = 0.04 respectively). Based on data from the six studies of stair ascent, the pooled mean difference (17.7 ms, 95% CI: 3.8 ms to 31.6 ms) suggested that there was a statistically significant delay in the onset of VMO during stair ascent, between in the AKP group and the control group. However, when the results of Boling et al [15] are excluded, based on their heterogeneity, the significance of this delay is no longer statistically significant (12.03 ms, 95% CI. -0.17 ms to 24.23 ms). During stair descent, the pooled estimate also indicated a statistically significant delayed VMO onset in AKP patients (pooled MD 30.25 ms, 95% CI: 16.68 ms to 43.81 ms) although again this is reduced when the results of Boling et al [15] are excluded (pooled MD 21.33 ms, 95% CI. 16.47 ms to 26.19 ms).

**Figure 3:**
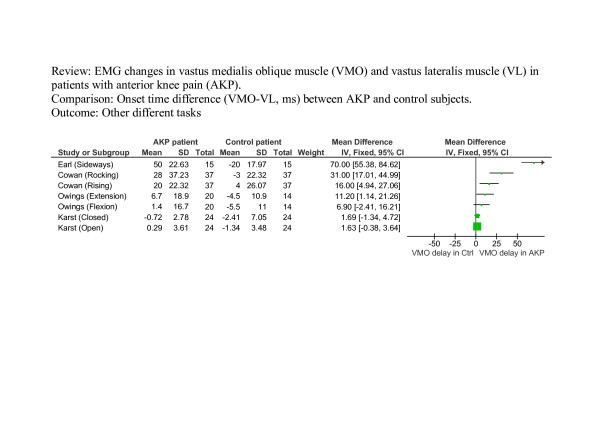
**Results from four studies that measured onset timing of the VMO and VL during different activities**. A negative VMO-VL value indicates VMO activation before VL activation. 2. Standard deviation for the study by Earl et al [17] is an estimate based on data from other studies. 3. Results from individual studies are not quantitatively combined since the onset timing was measured during different tasks.

The results from four studies which measured onset timing of the VMO and VL during functional activities other than stair ascent and descent are presented in Figure 3. Because the onset timing was measured during diverse tasks, the results were not quantitatively pooled. Data presented in Figure 3 indicates that the onset of VMO relative to VL tended to be delayed in the AKP group.

**Figure 4:**
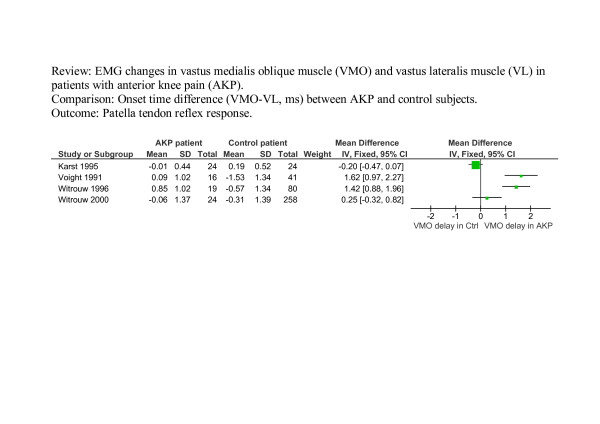
**The results for onset timing of VMO and VL during the patellar tendon reflex reaction**. A negative VMO-VL value indicates VMO activation before VL activation. 2. Data for mean was extracted from charts rather than the text for Voight and Weider [6]. 3. Standard deviation for Voight and Wieder [6] and Witrouw et al [18] is an estimate based on data from Karst and Willet [30] and Witrouw et al [13]. 4. Small study bias: Begg's test p = 0.73, Egger's test p = 0.11.

Figure 4 shows the results for onset timing of VMO and VL during the patella tendon reflex reaction. The heterogeneity across studies was substantial (I^2 ^= 93.4%, p < 0.0001). Although there was a tendency that onset of VMO was delayed in the AKP group compared to control subjects, the pooled mean difference (0.75 ms, 95% CI: -0.19 ms to 1.69 ms) by random-effects meta-analysis was not statistically significant (p = 0.12). The small study bias was not statistically significant (Begg's test p = 0.73, Egger's test p = 0.11).

Some studies provided insufficient raw data or necessary statistics in the text for meta-analysis [5,6,16-18,20,21]. For these studies the standard deviation was either estimated from graphs or expropriated from the data of other sources; details of which are provided in the legends of figures 2, 3, 4. The pooled weighted mean difference has therefore been calculated after excluding these studies to indicate the effect these studies may have had upon final results. For figure 2, excluding Brindle [21] (who did not report SD) and Cowan [5,16] (data was extracted from graphs), resulted in a pooled MD of 23.79 ms (95% CI -3.51 ms to 51.08 ms) for stair ascent with a heterogeneity I^2 ^of 92.5% (p < 0.001) and a pooled MD = 59.59 ms (95% CI -26.40 ms to 145.58 ms) for stair descent with a heterogeneity I^2 ^of 91.0% (p < 0.001). For figure 4, excluding Voight [6] (did not report SD and data was extracted from graphs) and Witrouw [18] (who did not report SD for VMO-VL), resulted in a pooled WMD of – 0.05 ms (95% CI -0.47 ms to 0.37 ms) and a heterogeneity I^2 ^of 48.1% (p = 0.16).

In summary, after excluding such studies, the trend observed in Figure 2 remains (the point estimates are further away from zero) but due to fewer studies and wider confidence intervals these are no longer statistically significant. The statistically non-significant difference in figure 4 is reduced to almost zero (-0.05) with a wider confidence interval.

## Discussion

### Critical appraisal

There was an observable trend for a delay in the activation of VMO relative to that of VL in AKP patients. However not all studies found evidence of this. The main finding was that of considerable heterogeneity both within groups, and between studies. In addition seven [5,6,16-18,20,21] of the fourteen studies provided insufficient documentation of the standard deviation from the mean within the text. Estimations were therefore made from graphs or extrapolated from other sources. Given the substantial heterogeneity of results both within and between groups, comparison of mean differences and estimated standard deviations therefore has limitations and may be seen as controversial. The observed trend of a delayed onset of VMO relative to VL in AKP patients may therefore be equally be due to chance and any interpretation otherwise should be viewed with caution.

Compared with large studies, small studies tend to produce results with large variation and can relatively easily be conducted and abandoned. A bias towards only publishing studies which detect a difference between groups is therefore greater in smaller studies [23]. Larger studies, irrespective of their outcome, are less vulnerable to publication bias. The testing of small study bias was statistically significant only across studies of stair descent (Figure 2). However, even when the testing of small study bias is statistically non-significant, the possibility of publication bias could not be ruled out because of the small number of studies included in meta-analyses (4 – 6 studies), and small sample sizes in the studies (10–47 in the AKP group).

Insufficient data to allow meta-analysis clearly affected the significance of results, which were reduced to insignificant when data estimated from graphs or expropriated from other sources was excluded. The trend for a delay in VMO recruitment identified for reflex response was almost reduced to zero and although the overall trend for stair ascent and descent rose, heterogeneity increased. Estimating standard deviation, from alternative sources, where considerable heterogeneity exists, has limitations and restricts the value of any inferences.

There were a number of issues in terms of study design which may have an impact on the validity of the included studies. None of the studies indicated whether the researcher was blinded to group allocation which may have led to potential bias in reporting and interpreting data. Repeatability of the results, although sometimes very good when reported, may be an issue in some studies, particularly when insufficient details were provided to allow reproduction of electrode positioning.

Inclusion and exclusion criteria was generally well presented with little variation between studies. With only three exceptions [6,19,46] explicit and detailed criteria for a diagnosis of AKP was presented. One possible source of heterogeneity was that seven studies excluded participants with a history of knee trauma, only including subjects with an insidious onset of AKP [5,14-16,20,21,30] whilst the remaining studies did not state such criteria. However even within this group of seven, there are considerable differences; for example Boling et al [15] demonstrate a greater range of results than for example Crossley et al [14], despite similar inclusion/exclusion criteria. This is surprising given the greater age range both within and between groups for the later [14].

Although not always explicitly stated, the majority of studies matched groups well in terms of age, either intentionally or by chance. In seven studies the mean age of subjects and controls was within a year and within three to four years in five studies. Owings et al [19] demonstrated the greatest discrepancy in age across groups, with the mean age of subjects on average ten years older than the control group. However, within their task grouping (see Figure 3), the difference in timing between subjects and controls was less significant than that of Cowan et al [20] or Earl et al [17], both of which were well matched for age. Boling et al [15] used the same source, and aged match subjects and controls successfully, yet demonstrated greater differences in timing between groups than any other study investigating ascending and descending stairs (see Figure 2). In only one study [14] was the mean age of subjects greater than controls and by 11 years. This study demonstrated earlier onset of VMO relative to VL and with a small standard deviation. Similarly, differences between studies in reflex response times cannot be attributed to differences in age between subjects and control groups. The age of participants clearly did not account for the heterogeneity between the studies.

Demographic details other than age may have affected the heterogeneity of results. Cowan et al [5,16,20] use physiotherapy students as controls. Physiotherapy students are often physically active, practicing new motor skills on a regular basis, and potentially aware of the VMO-VL debate, all of which may affect performance. Although well matched for age, most of the studies provide limited demographic data in terms of recreational pursuits and activity levels. Witrouw et al's [13] study was the only study to use one cohort of subjects, all of whom participated in 12–14 hours of sport weekly. Their results indicated little difference in timing between subjects with and without AKP. Earl et al [17] stated that as well as age, the fifteen recreational athletes in each group of their study were matched with regards to gender, height, weight and duration of exercise per week, and so this cannot account for the large differences between this group, compared to the other studies in Figure 4. However, recreational differences may have accounted for heterogeneity elsewhere.

Pain has been linked to changes in normal muscle recruitment in a number of musculoskeletal conditions [24-27]. All but one of the studies [15] investigating stair ascent and decent indicated the presence and intensity of pain during data collection using a ten point visual analogue scale (VAS). Boling et al [15] used this same scale to record pain during the week prior to data collection on a number of activities of which stair ascent and descent was one. Their mean pain scores were the highest for this grouping at 4.9 ± 2.3 and they demonstrate the greatest magnitude of difference between subjects and controls in VMO-VL recruitment (see Figure 2). However the influence of higher levels of pain during testing on VMO-VL results was not supported by Brindle et al's [21] study, in which only slightly lower VAS scores were recorded actually during testing, and where there was little difference between the groups in the opposite direction. In addition, Cowan et al [20], explicitly stated the absence of pain during rocking onto the heels, yet record the second highest difference between subjects and controls for this group of tasks (see Figure 3). The presence of pain during testing was not stated in any other studies. The heterogeneity of results in this review was not explained by the presence and intensity of pain during data collection.

Differences in the duration of AKP rather than the presence of pain during data collection may have influenced the heterogeneity of results between studies. When documented, the duration of AKP pain ranged from one week [17] to twelve years [5]. It is interesting to note that with the exception of Boling et al [15], these two studies demonstrated the largest difference between subject and control groups within their respective task groupings (see Figure 2 and 3). These extremes demonstrate that the duration of symptoms does not appear to be a factor contributing to the results in the reviewed studies.

None of the studies included in this review indicated whether participants were receiving physiotherapy or had done so in the past. Some physiotherapists seek to normalise aberrant muscle recruitment patterns with the intention of reducing pain [7,28,29]. The effectiveness of physiotherapy in achieving this specific target can only be assessed once a clearer definition of normal has been established.

### Factors affecting EMG data collection and analysis

The EMG studies included in this review investigated three types of muscle activation: reflex, voluntary closed kinetic chain, and voluntary open kinetic chain. From the literature, reflex onset times appear the most repeatable, and marked variability has been noted in voluntary EMG onset times [5,30], with open kinetic chain appearing the most variable [31]. This may have affected the heterogeneity seen in the results.

Many factors can affect EMG results, and methodological differences are frequently cited as reasons for the lack of agreement between studies. These factors can include electrode placement and orientation, data sampling rates, levels of smoothing or filtering, and onset determination methods. Readers are referred to articles such as Soderberg and Knutson [32] for more detail. Importantly, EMG onset times can be affected by the onset determination methods [32], and the level of EMG smoothing [33] (see Table 1). Various onset determination methods were used in the eighteen studies. Three of the four *reflex *studies use visual determination of EMG onset, and this has been shown to be highly repeatable [34]. The majority of studies investigating *voluntary *muscle activation determined EMG onset as the point at which the signal exceeded the mean resting "baseline" value, prior to activity, by more than a set number of standard deviations for a specified period of time. This is done to avoid type I errors, classifying the muscle as active when it is not. Both the number of standard deviations and period of time stated varied between studies, or were not stated. These factors are important for direct comparisons of individual muscle timing between studies. Of course, providing that an onset threshold was standardised for each study, there should have been relatively little affect on *between-group *differences in the relative activation times of VMO and VL, as any differences in threshold should have affected both muscles and both groups similarly. The level of smoothing was also important as excessive smoothing could reduce the ability to detect small timing differences that may be clinically relevant [33,35].

It is interesting that Boling et al [15] used the same EMG processing and analysis methods as in the studies by Cowan and Crossley et al [5,14,16], but obtained data that indicated far greater differences between the patient group and the control group. In addition, the magnitude of within-group standard deviation for the former is considerable and for the latter particularly small. This could be due to differences in the identification of the resting baseline window, which could have contained more or less muscle activity, and hence affected the onset threshold. Although all studies relating to stair ascent and descent reported EMG onset times relative to foot strike or stance phase, method of measurement varied. Boling et al [15] report EMG onset times to be pre-foot-strike (e.g. negative values are provided for independent VMO and VL onset timing) which contrasts with for example, McClinton et al [22] who evaluate onset times commencing at heel contact and Brindle et al [21], who evaluate onset times commencing with toe contact. The exact commencement of readings for "stance phase" for the Cowan and Crossley group [5,14,16] is at "foot contact". These differences are not always immediately apparent or explicit in the text.

Documentation of electrode positioning varied. Some studies provided references such Basmajian and Blumenstein [36], and Tully and Stillman [37] or provide anatomical landmarks, whilst others simply stated that electrodes were positioned over the muscle bellies of VMO and VL. This would make the replication of these studies challenging, whilst also making it difficult to directly compare results.

Reflex latency times are highly repeatable [34,38]. The differences found between the reflex reaction studies are therefore very interesting, and again, may be due to a number of reasons, including methodological differences in the stimulus used to deliver the patellar tendon tap, onset determination methods, or differences between participants such as symptom duration or height [30]. However, it has been noted that the range of reflex latencies displayed by Voight and Weider [6] was abnormally large [30], and included some values as low as 10 ms. These short latencies may be physiologically questionable [30], the normal range being approximately 16 to 30 ms [38], varying slightly with methodology, but importantly limited by the maximum human nerve conduction velocity of approximately 50 m/s. Whilst these very short latencies could possibly be due to time delays in the methodological set-up, they could have been movement artefacts on the EMG traces [30].

### Clinical relevance of VMO-VL timing differences

Whilst the findings of this review suggest a trend towards relatively delayed onset of the VMO when compared to the VL in the AKP population, the clinical significance of these findings is unclear. The differences in timing described are all relatively small, and it appears as yet unknown at what point such a difference may become clinically significant; although interestingly, Neptune et al [39] suggested that timing differences as low as 5 ms can elicit a biomechanical imbalance at the patellofemoral joint. It is notable however that this figure is lower than the standard error of the measurement reported by the Cowan and Crossley group [5], and marked within-subject variability in VMO – VL onset times in voluntary muscle activation have also been reported [3,30].

The between group and between subject variability recorded in each of the fourteen studies is considerable, and does not appear to be attributable to anything other than true variability between subjects. A comparison of mean group values, whether to reflect a trend or indicate a statistically significant finding, may be appropriate statistically, but is of questionable clinical relevance. The large variability between subjects in a given population, whether this be healthy or AKP patients, does not make it possible to make generalisations.

Differences in procedural and onset determination methods may account for the differences seen between studies in the timing of VMO and VL. However, as stated previously, this should have relatively little effect on the *relative activation times *of VMO and VL, the focus of this review. The possible sources of heterogeneity discussed between studies and summarised in Table 4, do not convincingly account for the differences in results across studies.

**Table 4:** Possible sources of heterogeneity

Methodology	Publication Bias Convenience rather than random sampling may not represent true population Lack of assessor blinding to group allocation Small sample size
Population characteristics	Intensity of AKP Duration of AKP Recreational pursuits Previous or concomitant physiotherapy Age range Previous knowledge pertaining to research question (i.e. population of physiotherapy students knowledge on VMO/VL debate and techniques to facilitate VMO
Procedural details	Differences between task Differences between similar tasks- e.g stair height, timing of task No of tests prior to data collection (learning effect/fatigue) Results recorded as single test or mean of several Electrode placement Data sampling rates EMG onset determination Levels of smoothing or filtering

Abbreviations:

AKP – anterior knee pain; EMG – electromyography; VL – vastus lateralis; VMO – vastus medialis oblique

Clinically, the relevance of any trends towards delayed VMO onset in the AKP population may be increased if there is evidence that therapies can favourably influence it. Some studies have demonstrated alterations in VMO-VL onset times in AKP patients following or during successful therapeutic interventions. Boling et al [15] and Cowan et al [40] demonstrated a significant delay in VMO activation prior to physiotherapy, and subsequently, following successful treatment with pain reduction, a significantly earlier VMO activation was observed. It has also been reported that therapeutic patella taping can improve VMO-VL onset time differences [16,41]. Indeed from a prospective study of 30 subjects, Witrouw [42] report that faster VMO onset times are a predictor of successful rehabilitation, although no primary data is provided. If the reported results of these small studies are transferable they could indicate that although normal timing is variable, enhancing VMO onset times by reducing any delay in activation may be associated with pain relief.

Clinically, it is interesting that the type of muscle contraction, i.e. reflex or voluntary, and closed or open kinetic chain, seems to influence variability, as stated in the Results section. This is probably due to differences in motor unit recruitment strategies between the contraction types, for example caused by differences in proprioceptive feedback and knee joint reaction forces between open and closed chain activities [31]. This may be a factor to be borne in mind in rehabilitation programmes aimed at treating aberrant muscle activation patterns in this pathology.

The overall tendency towards a delayed onset of VMO relative to VL in AKP patients was consistent for both voluntary functional and non-functional tasks, as well as to a lesser degree reflex response times. Despite this however, the heterogeneity across the studies was substantial and unexplained, and the questionable clinical significance of any such trend is highlighted by the fact that some asymptomatic individuals represented within the control groups demonstrate similar patterns of VMO to VL dysfunction – but do not experience AKP. One possibility is that patients with AKP are not a homogenous group, and that relative delay of the VMO represents just one of a variety of factors that may lead to this syndrome.

This review presented some limitations. Firstly, although all retrieved full text articles and some specialist journals were hand-searched, the majority of this review's search strategy was performed using computer databases. Accordingly relevant papers may have been missed by employing this method [43]. No attempt was made to identify unpublished work and grey literature (such as university theses and conference proceedings). As a result, publication bias may have influenced the results [23,44]. One foreign language paper [45] was identified by the search but excluded from our review. The quality of reporting compromised the validity of the included studies – in particular assessor blinding of group allocation and insufficient data to allow meta-analysis. This was therefore in part based on data extracted from graphical illustrations or expropriated from the data of other sources. Heterogeneity of results both within subject and control groups as well as between studies indicate that any trends identified should be interpreted with caution.

## Conclusion

The findings from this review are subject to substantial and unexplained heterogeneity, and the impact of publication bias and methodological flaws such as blinding to study allocation could not be ruled out. There were large variations within subject and control groups, as well as between studies. There was a trend for delayed onset of VMO relative to VL in subjects with AKP in comparison to those without. This was consistent for functional tasks such as ascending and descending stairs, stepping sideways and rocking onto the toes/heels, as well as less functional tasks such as isokinetic testing and reflex response times. However not all AKP patients demonstrate a VMO-VL dysfunction, and this is compounded by considerable normal physiological variability in the healthy population. Because of unexplained heterogeneity and methodological limitations, any inferences based on statistical analysis should be viewed with caution. The clinical and therapeutic significance of these findings based on the existing literature is therefore difficult to assess.

## Competing interests

The authors declare that they have no competing interests.

## Authors' contributions

RC co-coordinated the review, and contributed to the literature search, data extraction and drafting of the manuscript. TOS contributed to the literature search, data extraction and drafting of the manuscript. DS contributed to the literature search, data extraction and drafting of the manuscript. JD provided expertise on EMG analysis and contributed to drafting of the manuscript. SW contributed to the literature search and data extraction. FS provided expert advice on systematic reviewing, undertook meta-analysis and contributed to drafting of the manuscript. All authors read and approved the final manuscript.

## Pre-publication history

The pre-publication history for this paper can be accessed here:



## Supplementary Material

Additional file 1 Data extraction form. Table used to record study design, participant selection and population characteristics, methodology, results and relevant study limitations. Comparisons between reviewers were made for accuracy and interpretation. Click here for file

## References

[B1] Baquie P, Brukner P (1997). Injuries presenting to an Australian Sports Medicine Centre: a 12 month study. Clin J Sports Med.

[B2] McConnell J (1986). The management of chondromalacia patellae: a long term solution. Aust J Physiother.

[B3] Crossley KM, Bennell K, Green S, McConnell J (2001). A systematic review of physical interventions for patellofemoral pain syndrome. Clin J Sports Med.

[B4] McConnell J (1996). Management of patellofemoral problems. Man Ther.

[B5] Cowan S, Bennell K, Hodges P, Crossley K, McConnell J (2001). Delayed onset of electromyographic activity of vastus medialis obliquus relative to vastus lateralis in subjects with patellofemoral pain syndrome. Arch Phys Med Rehabil.

[B6] Voight M, Wieder D (1991). Comparative reflex response times of vastus medialis obliquus and vastus lateralis in normal subjects and subjects with extensor mechanism dysfunction. Am J Sports Med.

[B7] Powers C (1998). Rehabilitation of patellofemoral joint disorders: a critical review. J Orthop Sports Phys Ther.

[B8] Thomee R, Augustsson J, Karlsson J (1999). Patellofemoral pain syndrome. Sports Med.

[B9] Pigott TD, Cooper H, Hedges LV (1994). Methods for handling missing data in research synthesis. The handbook of research synthesis.

[B10] Higgins JP, Thompson SG, Deeks JJ, Altman DG (2003). Measuring inconsistency in meta-analyses. BMJ.

[B11] Begg CB, Mazumdar M (1994). Operating characteristics of a rank correlation test for publication bias. Biometrics.

[B12] Egger M, Davey-Smith G, Schneider M, Minder C (1997). Bias in meta-analysis detected by a simple, graphical test. BMJ.

[B13] Witvrouw E, Lysen R, Bellemans J, Cambier D, Vanderstraeten G (2000). Intrinsic risk factors for the development of anterior knee pain in an athletic population. A two-year prospective study. Am J Sports Med.

[B14] Crossley KM, Cowan SM, Bennell KL, McConnell J (2004). Knee flexion during stair ambulation is altered in individuals with patellofemoral pain. J Orthop Res.

[B15] Boling MC, Bolgla LA, Mattacola CG, Uhl TL, Hosey RG (2006). Outcomes of a weight-bearing rehabilitation program for patients diagnosed with patellofemoral pain syndrome. Arch Phys Med Rehabil.

[B16] Cowan SM, Bennell KL, Hodges PW (2002). Therapeutic patellar taping changes the timing of vasti muscle activation in people with patellofemoral pain syndrome. Clin J Sports Med.

[B17] Earl JE, Hertel J, Denegar CR (2005). Patterns of dynamic malalignment, muscle activation, joint motion, and patellofemoral-pain syndrome. J Sports Rehabil.

[B18] Witvrouw E, Sneyers C, Lysen R, Victor J, Bellemans J (1996). Reflex response times of vastis medialis oblique and vastis lateralis in normal subjects and in subjects with patellofemoral pain syndrome. J Orthop Sports Phys Ther.

[B19] Owings TM, Grabiner MD (2002). Motor control of the vastis medialis oblique and vastis lateralis muscles is disrupted during eccentric contractions in subjects with patellofemoral pain. Am J Sports Med.

[B20] Cowan SM, Hodges PW, Bennell KL, Crossley KM (2002). Altered vastii recruitment when people with patellofemoral pain syndrome complete a postural task. Arch Phys Med Rehabil.

[B21] Brindle TJ, Mattacola C, McCrory J (2003). Electromyographic changes in the gluteus medius during stair ascent and descent in subjects with anterior knee pain. Knee Surg Sports Traumatol Arthrosc.

[B22] McClinton S, Donatell G, Weir J, Heiderscheit B (2007). Influence of step height on quadriceps onset timing and activation during stair ascent in individuals wit patellofemoral pain syndrome. J Orthop Sports Phys Ther.

[B23] Song F, Eastwood AJ, Gilbody S, Duley L, Sutton AJ (2000). Publication and related biases. Health Technol Assess.

[B24] Steenbrink F, de Groot JH, Veeger HEJ, Meskers CGM, van de Sande MAJ, Rozing PM (2006). Pathological muscle activation patterns in patients with massive rotator cuff tears, with and without subacromial anaesthetics. Man Ther.

[B25] Vogt L, Pfeifer K, Banzer W (2003). Neuromuscular control of walking with chronic low-back pain. Man Ther.

[B26] Le Pera D, Graven-Nielsen T, Valeriani M, Oliviero A, Di Lazzaro V, Tonali PA, Arendt-Nielsen L (2001). Inhibition of motor system excitability at cortical and spinal level by tonic muscle pain. Clinical Neurophysiology.

[B27] Rutherford OM, Jones DA, Newham DJ (1986). Clinical and experimental application of the percutaneous twitch superimposition technique for the study of human muscle activation. J Neurol Neurosurg Psychiatry.

[B28] Powers CM, Landel R, Perry J (1996). Timing and intensity of vastis muscle activity during functional activities in subjects with and without patellofemoral pain. Phys Ther.

[B29] Crossley KM, Cowan SM, Bennell KL, McConnell J (2000). Patella taping: is clinical success supported by scientific evidence?. Manual Therapy.

[B30] Karst GM, Willett GM (1995). Onset timing of electromyographic activity in the vastus medialis oblique and vastus lateralis muscles in subjects with and without patellofemoral pain syndrome. Phys Ther.

[B31] Stensdotter A-K, Hodges PW, Mellor R, Sundelin G, Hager-Ross C (2003). Quadriceps activation in closed and in open kinetic chain exercise. Med Sci Sports Exerc.

[B32] Soderberg GL, Knutson LM (2000). A guide for use and interpretation of kinesiologic electromyographic data. Phys Ther.

[B33] Merletti R (1999). Standards for reporting EMG data. J Electromyogr Kinesiol.

[B34] Dixon J, Howe TE, Kent JR, Whittaker VJ (2004). VMO-VL reflex latency difference between quadriceps components in osteoarthritic and asymptomatic knees. Adv Physiother.

[B35] Karst GM (1998). EMG onset timing. Phys Ther.

[B36] Basmajian J, Blumenstein R (1980). Electrode placement in EMG biofeedback.

[B37] Tully EA, Stillman BC (1997). Computer aided video analysis of vertebrofemoral motion during toe touching in healthy subjects. Arch Phys Med Rehabil.

[B38] Struys MA, Jonkman EJ, Strijers RL (1997). Measurement of patellar and ankle tendon reflexes in normal subjects. Electromyogr Clin Neurophysiol.

[B39] Neptune RR, Wright IC, van den Bogert AJ (2000). The influence of orthotic devices and vastus medialis strength and timing on patellofemoral loads during running. Clin Biomech.

[B40] Cowan SM, Bennell KL, Hodges PW, Crossley KM, McConnell J (2003). Simultaneous feedforward recruitment of the vasti in untrained postural tasks can be restored by physical therapy. J Orthopaedic Research.

[B41] Gilleard W, McConnell J, Parsons D (1998). The effect of patella taping on the onset of vastus medialis obliquus and vastus lateralis muscle activity in persons with patellofemoral pain. Phys Ther.

[B42] Witvrouw E, Lysen R, Bellemans J, Cambier D, Cools A, Danneels L, Bourgois J (2002). Which risk factors predict outcome in the treatment program of anterior knee pain?. Scand J Med Sci Sports.

[B43] Colville-Stewart S, Tarling M, Croft L (2002). How to do a literature search. The essential researcher's handbook For nurses and healthcare professionals.

[B44] Petrie A, Sabin S (2000). Medical statistics at a glance.

[B45] Aguggia M, Gilli M, Febbraro A, Riccio A, Viglino C, Losana A, Carando S, Rossi P (1997). Comparative electromyographic study of the vastus medialis muscle and vastus lateralis muscle in dysfunctions of the knee extensor mechanism. Minerva Ortopedica e Traumatologica.

[B46] Morrish GM, Woledge RC (1997). A comparison of the activation of muscles moving the patella in normal subjects and in patients with chronic patellofemoral problems. Scand J Rehabil Med.

