# Supplementary material for: The relative timing of VMO and VL in the aetiology of anterior knee pain: a systematic review and meta-analysis
Source: BMC Musculoskelet Disord. 2008 May 1;9:64. doi: 10.1186/1471-2474-9-64 (PMC2386790; doi:10.1186/1471-2474-9-64)
Supplement: Additional file 1 — Data extraction form. Table used to record study design, participant selection and population characteristics, methodology, results and relevant study limitations. Comparisons between reviewers were made for accuracy and interpretation. [file 1471-2474-9-64-S1.doc]

Additional file 1: Data extraction form

| Author (year)_________ Reviewer: ____ Date of review:___________ |
| --- |
| **Title and journal:** |
| **Study design:**  Comparative observational / longitudinal |
| **Participant Selection**  Subject selection  Convenience / other (state which)/ not stated  Subject profile  Geographic area / not stated  Hospital / sports club / not stated  Professional athletes / recreational athletes / non athletes / school age/ not stated  Control selection  Convenience / matched / other (state which) / not stated / not applicable (if longitudinal study)  Control profile (not applicable if longitudinal study)  Geographic area/ not stated  Hospital / sports club / not stated  Professional athletes / recreational athletes / non athletes / school age/ not stated   |  | N at start of study | N at end of study (and included in stats) | Age  (mean, SD, range) | Height | Gender  M/F | | --- | --- | --- | --- | --- | --- | | Subjects - AKP |  |  |  |  |  | | Controls |  |  |  |  |  | | Differences between groups.  State p value. |  |  |  |  |  |  | Inclusion Criteria (Duration, co morbidity) and exclusion criteria | | | | --- | --- | --- | | Subjects - AKP | Controls |  | |  |  |  |   Duration of symptoms (if stated mean and range or SD)?  Justification for sample size? Yes / no (If stated was this for timing and based on what clinical difference?)  Have they had or are they having physio and if so does it involve Quads work? Yes (state details) / no / not stated |
| **Methodology**  Method of testing  Patella hammer, isometric, functional (state details)  State what was measured, no of measurements and which was used – mean or best of etc  Position of testing  ie high sitting, hip/knee at 90 degrees of F  Electrode positioning reproducible? Yes / no / not sure  Electrode positioning?  Over muscle bellies / over VM/VL/VMO motor point / anatomical landmarks (provide details) / reference (state details)  Apparatus details  (As stated in paper). Not stated  Was the reliability of TIMING stated? Yes / no (reliability if stated)  Blinding of testers to group allocation? Yes / no / not stated  Number of examiners? Not stated  Was AKP present during the test? Yes / no / not stated  Statistical test used (state) |
| **Results:** What did they find? **Indicate source of information if other than text**   | Study group | Muscle/side tested | Mean | SD or SE | p value | | --- | --- | --- | --- | --- | | AKP | VMO (R) or (S)  VMO (L)or (A) |  |  |  | | VL (R) or (S)  VL (L) or (A) |  |  |  | | VMO-VL (R) or (S)  VMO-VL (L) or (A) |  |  |  | | Control | VMO (R)  VMO (L) |  |  |  | | VL (R)  VL (L) |  |  |  | | VMO-VL (R)  VMO-VL (L) |  |  |  | | Differences b’tn groups | State direction of AKP group relative to normals (control) |  |  |  |   Key for (R) and (L) side or symptomatic (S) versus asymptomatic (A) – indicate which. |
| Is data demonstrated via a distribution curve? Yes / no |
| **Clinical interpretation**  Was any indication of a clinical meaningful change provided? Yes / no |
| **Key finding/clinical bottom line/direction of difference** |
| **Any other commentary/Follow up questions:** |
